# Effects of altitudes on secondary metabolite contents of *Origanum majorana* L.

**DOI:** 10.1038/s41598-023-37909-0

**Published:** 2023-07-04

**Authors:** Emel Karaca Öner, Meryem Yeşil

**Affiliations:** Plant and Animal Production Department, Technical Sciences Vocational School, Ordu University , Ordu, Turkey

**Keywords:** Plant breeding, Plant ecology, Secondary metabolism

## Abstract

Altitude is an important ecological factor that significantly affects essential oil content, yield and composition. In this study, conducted to examine the effects of altitude on essential oil content and composition of *O. majorana*, plant samples were collected from the southern region of Türkiye at the beginning of flowering period from seven different altitudes (766 m, 890 m, 968 m, 1079 m, 1180 m, 1261 m and 1387 m) at 100 m intervals. The highest percentage of essential oil (6.50%) obtained by hydro-distillation was determined at 766 m altitudes. The GC–MS analyses revealed that low altitude affected some essential oil components positively. The linalool ratio, which is the major component of the essential oil of *O. majorana* species, was the highest at 766 m (79.84%) altitudes. Borneol, linalool oxide, trans-linalool oxide, caryophyllene, *a*-humulene, germacrene-D and bicyclogermacrene components yielded high values at 890 m altitudes. Thymol and α-terpineol, which have an important place in the essential oil composition, increased at 1180 m altitudes; *a*-terpinene, *cis*-sabinene hydrate, terpinene-4-ol and carvacrol increased at 1387 m altitudes.

## Introduction

Thyme is a pungent herb of Lamiaceae family and has several species used for medicinal and aromatic purposes. In Türkiye flora, several species of *Origanum, Thymus, Satureja, Thymbra* and *Coridothymus* genera are known as thyme^1,2^. It has a great morphological and chemical diversity and has a natural widespread with 42 species in Eurasia and North Africa^3,4^. It is represented by 22 species in Türkiye flora and endemism rate is around 63%^5^.

Thyme has a great economic importance. It is generally consumed as a spice and in small amounts in the form of thyme tea, thyme oil or thyme juice. Since thyme contains essential oils with highly strong antimicrobial and antioxidant effects, it is of great importance as an additive in foods, perfumes, cosmetics, medicines, lotions, soaps and toothpastes^6–8^. Thyme essential oil is composed of terpineol, linalool, carvacrol, cymol, thymol, p-cymene and borneol. Thymol and carvacrol are responsible for the scent of the plant. These substances constitute the main components of thyme essential oil. Carvacrol is mostly found in the essential oil of *Thymbra*, *Origanum* and *Satureja* species and thymol is found in higher proportions in the essential oil of *Thymus* species^9^.

*O. majorana* species belonging to the *Origanum* genus, also known as marjoram, is widely used as a spice, also used in folk medicine for the treatment of diseases such as asthma, indigestion, headache and rheumatism^10^; antibacterial^11^ and antiviral^12^ effects have also been reported. Its main components were reported as sabinene linalyl acetate and cis-sabinene by^13^; as carvacrol (65%), thymol (4%), terpinene-4-ol (31.15%), *cis*-sabinene hydrate (15.76%), p-cymene (6.83%), sabinene (6.91%), *trans*-sabinene hydrate (3.86%) and *a*-terpineol (3.71%) by^14^ as terpinene-4-ol (38.40%), cis-sabinene hydrate (14.95%), *p*-cymene (7.01%) and *a*-terpineol (4.88%) by^15^.

Chemical composition of *Origanum* species is largely designated by different chemotypes, geographical origins and harvest times^16,17^. Although the effect of altitude on essential oil components of *Origanum* species has been investigated by some researchers^4,18–20^, no comprehensive study has been found in *O. majarona* species. Secondary metabolites are not solely under the control of genetic factors. Biotic and abiotic factors that alter essential oil composition and rates include altitude, temperature, precipitation, humidity, wind exposure, light intensity, plant vegetative composition and plant growth periods^4,19,21–26^.

This study was conducted to investigate the effects of natural altitudes on essential oil content and composition of *O. majorana* species.

## Materials and methods

### Plant material

**Figure 1:**
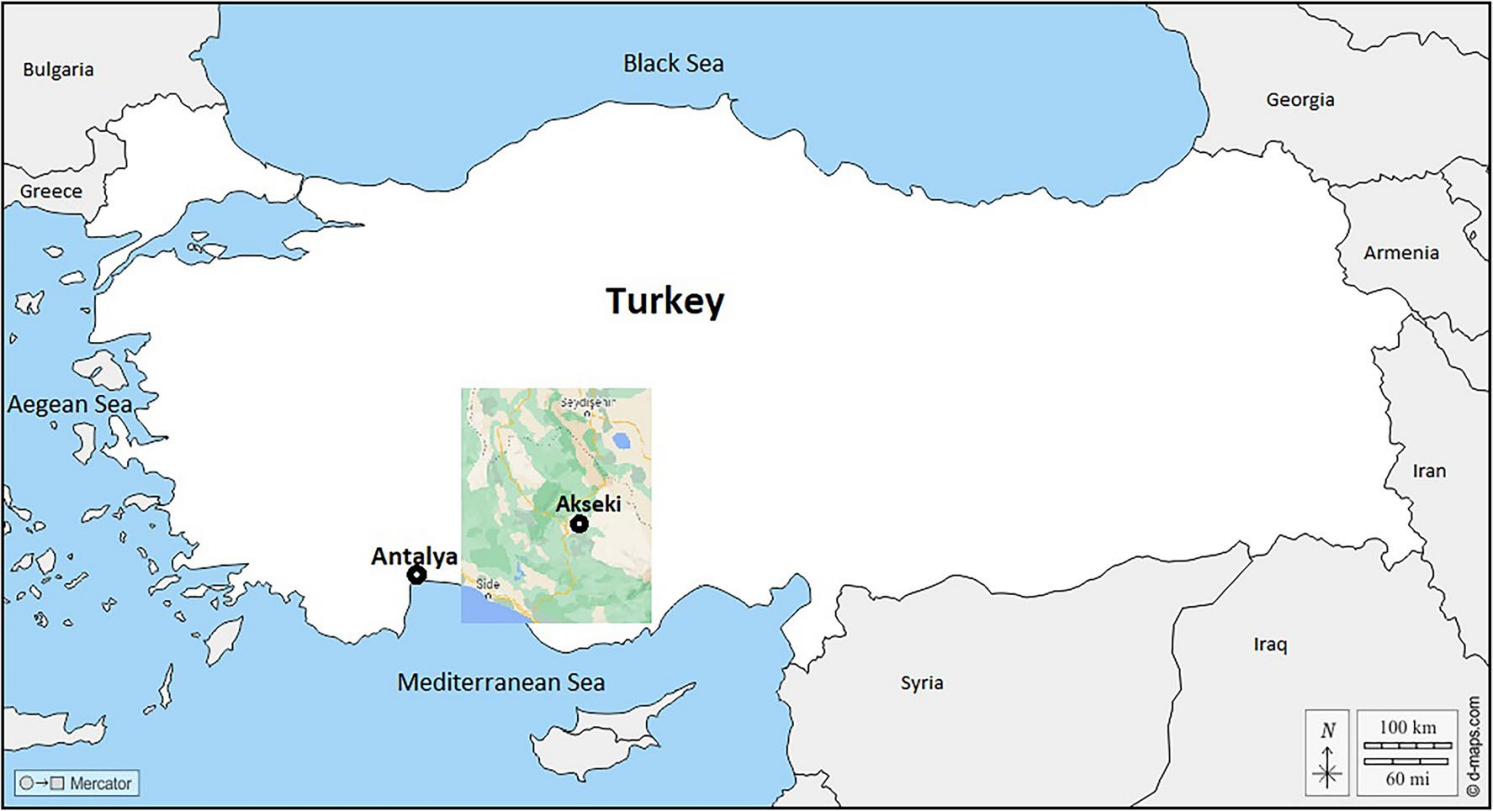
Türkiye map showing “Akseki” upland in territory of Antalya province.

Samples were collected from Antalya province, located in southwest of Türkiye between the 36° 07′–37° 20′ north latitudes and 29° 20′–32° 35′ east longitudes^27^. In the Akseki region of Antalya province, where plant samples were collected, the average temperature for 2022 was 26.33 °C, the average relative humidity was 50.89%, and the average precipitation was 86.1 mm. The *O. majorana* samples were collected from natural flora of Akseki district at seven different altitudes in 100 m intervals (766 m, 890 m, 968 m, 1079 m, 1180 m, 1261 m and 1387 m). Samples were collected at the beginning of 2022 vegetation period between the hours 9:00–11:00 a.m. (Fig. 1).

Dr. Sevda Türkiş of the biology department at Türkiye's Ordu University's Faculty of Education and Sciences identified the species. The Technical Sciences Vocational School Plant and Animal Production Department has a herbarium where voucher specimens have been placed. At 9:00 am and 11:00 pm, the tops of two thirds of the plants were plucked. The plant materials were dried at room temperature (25 ± 2 °C) before being tested for polar compounds using GC–MS. We confirm that all methods were performed in accordance with the relevant guidelines and regulations by including a statement in the methods section. Table 1 displays the voucher numbers and geographic information for plant-growing ecosystems.

**Table 1:** The locations where the populations of *O. majorana* were collected for study.

Voucher no	Populations	Altitude (m)	Coordinates
OUTBMYO # 1/1*	Akseki Antalya, Türkiye	766	N 37 80 51 E 41 04 185
OUTBMYO # 1/2	Akseki Antalya, Türkiye	890	N 38 42 62 E 41 03 465
OUTBMYO # 1/3	Akseki Antalya, Türkiye	968	N 38 53 33 E 41 04 225
OUTBMYO # 1/4	Akseki Antalya, Türkiye	1079	N 38 81 18 E 41 11 960
OUTBMYO # 1/5	Akseki Antalya, Türkiye	1180	N 38 65 67 E 41 02 756
OUTBMYO # 1/6	Akseki Antalya, Türkiye	1261	N 38 80 27 E 41 07 617
OUTBMYO # 1/7	Akseki Antalya, Türkiye	1387	N 39 61 33 E 40 84 954

Ordu University, Technical Sciences Vocational School, Plant and Animal Production Department.

### Essential oil extraction

Leaf-stem separation was made on samples collected from each altitude. Leaves were dried in shade and ground. Ground leaves (100 g in each replicates) were subjected to distillation process for 3 h in a Clevenger apparatus. Essential oil quantities were determined with the aid of a graduated tube and transferred to vials and stored at + 4 °C until the relevant analyses.

### Analysis of the essential oil

Essential oil components were determined by GC–MS (Agilent Technologies) instrument. Helium was used as carrier gas, GC–MS parameters are presented below.

Column: DB-WAX (60 m × 0.25 mm i.d., x film thickness 0.25 pm)

Dedektör: Agilent Technologies 5977A MS

Injection temp: 250 °C

Injection mode: Split

Split ratio: 150

Flow control mode: Pressure

Pressure: 80 kPa

Split ratio: 25

Column temperature programmed: 40 °C 2 min, 4 °C/min 240 °C 3 min

Ion source temp: 200 °C

Interface temp: 250 °C

Start time: 0 min; end time: 55 min

Acquisition mode: Scan

Start *m/z*: 40; end *m/z*: 4000

W9N11.L (Wiley Registry^®^ of Mass Spectral Data 9th edition + Nist 11th Edition Library) and MPW2011.L (Mass Spectral Library of Drugs, Poisons, pesticides, Pollutans and their Metabolites 2011 Library) libraries were used for identification of essential oil components.

### Statistical analysis

Experiments were conducted in randomized plots design with three replications. Experimental data were subjected to analysis of variance with the use of SAS-JMP 13.0 statistical software and significant means were compared with the use of LSD multiple comparison test.

## Result and discussion

### Essentional oil ratio

The essential oil content and composition of the plant part to be used as a drug in medicinal and aromatic plants are the most important criteria that designate the quality of the product^28,29^. Previous studies have revealed that genetic structure of the plant material used in production and ecological conditions of the region where the production is made had significant effects on essential oil ratios of aromatic plants^30–34^.

Effects of altitude on essential oil ratios are provided in Table 2 and altitude differences were found to be significant (p < 0.001). Essential oil ratios of the samples varied between 4.67 and 6.50%, with the highest value (6.50%) from 766 m altitudes and the lowest value (4.67%) from 1079 m altitudes. Essential oil ratios decreased with increasing altitudes, but such a decrease was placed into the same statistical group, except for the altitudes of 800–900 m (5.43%) and 1079 m (4.67%). Altitude-dependent differences in essential oil ratios were mainly attributed to ecological factors that change with altitudes^35^. Essential oil ratios of medicinal and aromatic plants usually vary with the climate components such as temperature, precipitation, relative humidity, day light hours, light intensity, soil conditions and development period^31,36–38^. The highest essential oil ratio of 766 m altitudes could be attributed to greater air temperature and relative humidity than the other altitudes (Table 2).

**Table 2:** Descriptive statistics for essential oil ratios of *O. majarona* species.

Altitude (m)	Essential oil ratio (%)
766	6.50 ± 0.23a
890	5.43 ± 0.46bc
968	6.15 ± 0.46ab
1079	4.67 ± 0.43c
1180	5.85 ± 0.08ab
1261	5.68 ± 0.28ab
1387	5.73 ± 0.25ab
p: 0.0004***	

Difference in means indicated with different letter in the same column is significant (***p < 0.001).

The factors that alter essential oil ratios of aromatic plants should be well-comprehended^34,39^, since pharmacopoeias specify the minimum essential oil ratios that aromatic plants must contain in order to be accepted as drugs and used as medicine and drugs containing essential oils below these ratios are not allowed to be used as medicine^40^.

### Essential oil components

Majority of the terpenes produced by plants are products of secondary metabolism and play an important role also in primary metabolism. These terpenoid structures, with their highly complex chemical structures, show great variation within and between the species and constitute the natural defense system of plants by damaging the other living things^41^.

The GC–MS analysis revealed 69–126 terpenoid structures for essential oils of *O. majorana* samples collected from different altitudes. Terpenoid ratios varied between 0.01 and 79.84%. The differences in ratios of all components with altitude were found to be significant (p < 0.001). Analyses on oregano, thyme and marjoram species revealed that mono and sesquiterpenes were the major components of essential oils of these species^42^. In this study, the ratio of oxygenated monoterpenes that form the essential oil and the terpenoid structure of *O. majorana* plants collected from different altitudes ranged between 65.69 and 84.62% and took the first place in total amount of essential oil. Oxygenated monoterpene ratios mathematically decreased with increasing altitudes, but there was no linear increase or decrease in oxygenated sesquiterpene ratios. Similar findings were also reported by^43^. Oxygenated monoterpenes were respectively followed by phenolic monoterpene (3.29–25.91%), sesquiterpene hydrocarbons (2.92–5.94%), oxygenated sesquiterpene (0.92–1.63%) and monoterpenes hydrocarbons (0.04–0.17%) (Table 3).

**Table 3:** Chemical composition of *O. majorana* essential oil.

Components	RT	f	766	890	968	1079	1180	1261	1387
Monoterpenes hydrocarbons			**0.06**	**0.04**	**0.08**	**0.07**	**0.11**	**0.13**	**0.17**
α-Terpinene	17.006	0.0001***	0.06 ± 0.006^de^	0.04 ± 0.0^e^	0.08 ± 0.0^ cd^	0.07 ± 0.01^de^	0.11 ± 0.01^bc^	0.13 ± 0.03^b^	0.17 ± 0.02^a^
Oxygenated monoterpene			**83.13**	**82.93**	**84.62**	**84.11**	**71.43**	**75.90**	**65.69**
Linalool	29.581	0.0001***	79.84 ± 1.10^a^	77.75 ± 3.03^ab^	79.68 ± 2.11^ab^	79.20 ± 0.63^ab^	67.62 ± 0.13^ cd^	71.53 ± 4.21^bc^	60.86 ± 5.23^d^
α-Terpineol	33.956	0.0001***	0.55 ± 0.02^d^	0.63 ± 0.05^ cd^	0.76 ± 0.05^bc^	0.88 ± 0.13^ab^	1.04 ± 0.00^a^	0.90 ± 0.02^ab^	0.86 ± 0.09^ab^
Borneol	34.248	0.0001***	0.32 ± 0.02^e^	0.97 ± 0.04^a^	0.83 ± 0.04^abc^	0.87 ± 0.04^ab^	0.65 ± 0.01^d^	0.77 ± 0.01^bcd^	0.69 ± 0.15^ cd^
*Cis*-sabinene hydrate	26.810	0.0002***	0.27 ± 0.01^b^	0.28 ± 0.02^b^	0.30 ± 0.03^b^	0.44 ± 0.07^b^	0.39 ± 0.0^b^	0.43 ± 0.03^b^	0.82 ± 0.26^a^
Terpinen 4-ol	31.298	0.0001***	0.22 ± 0.01^d^	0.24 ± 0.03^d^	0.34 ± 0.04^bc^	0.28 ± 0.02^ cd^	0.42 ± 0.00^b^	0.43 ± 0.02^b^	0.55 ± 0.07^a^
1,8-Cineole	18.173	0.0010***	0.08 ± 0.0^d^	0.31 ± 0.0^ab^	0.33 ± 0.0^a^	0.14 ± 0.0^bcd^	0.13 ± 0.0^ cd^	0.25 ± 0.0^a^'^d^	0.28 ± 0.0^abc^
Linalool oxide	26.129	0.0007***	0.75 ± 0.02^bc^	1.28 ± 0.05^a^	1.08 ± 0.04^ab^	0.99 ± 0.07^abc^	0.63 ± 0.16^c^	0.74 ± 0.23^bc^	0.82 ± 0.07^bc^
*Trans*-linalool oxide	27.069	0.0001***	1.10 ± 0.03^ab^	1.47 ± 0.01^a^	1.30 ± 0.01^a^	1.31 ± 0.31^a^	0.55 ± 0.01^c^	0.85 ± 0.26^bc^	0.81 ± 0.09^bc^
Phenolic monoterpene			**3.29**	**4.99**	**5.76**	**3.70**	**19.93**	**14.89**	**25.91**
Thymol	46.255	0.0001***	0.32 ± 0.02^c^	0.44 ± 0.11^c^	0.49 ± 0.17^c^	0.45 ± 0.09^c^	6.28 ± 0.03^a^	0.99 ± 0.53^c^	4.41 ± 1.15^b^
Carvacrol	47.000	0.0001***	2.97 ± 0.13^c^	4.55 ± 1.90^c^	5.27 ± 1.54^c^	3.25 ± 0.79^c^	13.65 ± 0.05^b^	13.90 ± 3.95^b^	21.50 ± 5.25^a^
Sesquiterpene hydrocarbons			**4.82**	**5.94**	**4.44**	**4.89**	**4.78**	**4.01**	**2.92**
Caryophyllene	31.541	0.0001***	2.76 ± 0.06^a^	2.78 ± 0.10^a^	2.41 ± 0.0^ab^	2.42 ± 0.23^ab^	2.29 ± 0.01^b^	1.61 ± 0.03^c^	1.80 ± 0.25^c^
α-Humulene	33.648	0.0006***	0.15 ± 0.01^bc^	0.21 ± 0.02^a^	0.15 ± 0.01^bc^	0.16 ± 0.02^abc^	0.13 ± 0.00^bc^	0.16 ± 0.04^ab^	0.11 ± 0.02^c^
Germacrene-D	34.766	0.0001***	0.18 ± 0.01^bc^	0.31 ± 0.02^a^	0.20 ± 0.01^bc^	0.32 ± 0.01^a^	0.22 ± 0.01^b^	0.29 ± 0.04^a^	0.17 ± 0.0^c^
Bicyclogermacrene	35.463	0.0001***	1.73 ± 0.04^c^	2.64 ± 0.17^a^	1.68 ± 0.05^c^	1.99 ± 0.17^bc^	2.14 ± 0.01^b^	1.95 ± 0.26^bc^	0.84 ± 0.02^d^
Oxygenated sesquiterpene			**1.11**	**1.27**	**1.32**	**1.63**	**0.92**	**1.20**	**1.18**
Caryophyllene oxide	42.301	0.0001***	0.26 ± 0.02^b^	0.33 ± 0.03^ab^	0.31 ± 0.03^ab^	0.35 ± 0.01^a^	0.15 ± 0.01^c^	0.18 ± 0.040^c^	0.30 ± 0.04^ab^
Sapthulenol	45.380	0.0067***	0.85 ± 0.22^b^	0.94 ± 0.15^ab^	1.01 ± 0.14^ab^	1.28 ± 0.08^a^	0.77 ± 0.02^b^	1.02 ± 0.12^ab^	0.88 ± 0.09^b^
Others			**7.59**	**4.83**	**3.78**	**5.60**	**2.83**	**3.87**	**4.13**
Total (%)			**100**	**100**	**100**	**100**	**100**	**100**	**100**

In the same row, the difference between the component ratios without a common letter is significant (***p < 0.001).

Values in bold refer to the percentage distribution of components.

In studies on essential oil composition of *O. majorana*, major components of essential oils were reported as terpinene-4-ol and sabinene hydrate by^43^ and as thymol and carvacrol by^14^. In this study, the major component was linalool (60.86–79.84%), an oxygenated monoterpene. Linalool is an essential oil component used in soaps, cosmetics, perfumes, cleaning products, food preservatives, herbicides and insecticides. Linalool, which has strong antimicrobial and antioxidant properties, is the essential oil component of many medicinal and aromatic plants belonging to Lamiaceae, Lauraceae and Rutaceae families^44^.

Both within the oxygenated monoterpene group components and among the other components that make up the essential oil, linalool ratios varied between 79.84% at 766 m altitudes and 60.86% at 1387 m altitudes. These values were the highest values among the total essential oil components. Linalool ratios decreased significantly (p < 0.001) with increasing altitudes. Similarly, greater linalool oxide (890 m: 1.28%) and trans-linalool oxide (890 m: 1.47%, 968 m: 1.30%, 1079 m: 1.31%) ratios were seen at lower altitudes. Linalool and linalool oxide ratios generally decrease with decreasing temperatures of high altitudes. Previous researchers emphasized that increase in phenolic components was positively affected by regional high temperatures^42,45–48^. The cis-sabinene hydrate (0.82%) and terpinene-4-ol (0.55%) ratios were positively affected by increasing altitudes (p < 0.001) and the highest values of both were seen at 1387 m altitudes. The a-terpineol ratio of 0.55% at 766 m altitudes increased to 1.04% at 1079 m altitudes and borneol ratio of 0.32% at 766 m increased to 0.97% at 890 m altitudes and the difference was found to be significant (p < 0.001). The lowest 1,8-cineol ratio was determined as 0.08% at 766 m and the highest ratio as 0.33% at 968 m altitudes. Researchers have reported that differences in temperature, relative humidity, wind speed and light intensity as you go to higher altitudes above sea level will change the physiological reactions of the plant and thus will create variations in composition of secondary metabolites^25,49,50^.

Thymol content within total essential oil was measured as 0.32% at 766 m, 0.44% at 890 m, 0.49% at 968 m, 0.45% at 1079 m and 0.99% at 1261 m altitudes. These values were statistically placed into the same group. Thymol ratio was 6.28% at 1180 m altitudes and 4.41% thymol was detected at 1387 m, which is the highest altitude and the the first place statistically.

High levels of carvacrol are encountered in *Origanum, Thymbra, Cordiothymus, Satureja* and *Lippia* species. Increasing carvacrol ratios were seen with increasing altitudes and the greatest value (21.50%) was seen at 1387 m altitudes. The increase in both thymol and carvacrol ratio with increasing altitude may be due to the prolongation of the growth period and the emergence of sufficient vegetation period for the conversion of intermediate components to these two components^42^. At high altitudes, plants are exposed to higher light intensity and lower average temperature. Therefore, plants at high altitudes have developed a protection mechanism against climate-induced damages. Plants adapted to high altitudes perform photosynthesis more effectively at low temperatures^51–53^. Previous studies indicated that carvacrol ratios increased with increasing altitudes^54^ and carvacrol ratios increased under stress conditions arising from environmental conditions^55^.

The *a*-terpinene represents monoterpenes hydrocarbons and the least encountered in monoterpene structures of *O. majarona* essential oil. The greatest value (0.17%) was seen at 1387 m altitudes. Previous researchers also reported that climate and environmental factors affected the essential oil composition^20,56,57^.

Caryophyllene is also a constituent of sesquiterpene hydrocarbon structure. Caryophyllene ratio was determined as 2.76% at 766 m and 2.78% at 890 m altitudes and they were placed into the same statistical group. Caryophyllene ratios decreased with increasing altitudes and decreased to 1.61% at 1261 m and 1.80% at 1387 m altitudes. The α-humulene (0.21%) and bicyclogermacrene (2.64%) ratios had the highest values at altitude of 890 m altitudes. At the highest altitudes (1387 m), a-humulene ratio decreased to 0.11% and bicyclogermacrene to 0.84%. Like the other sesquiterpene hydrocarbons, the lowest germacrene-D ratio (0.17) was seen at 1387 m altitudes and germacrene-D ratios at 890 m (0.31%), 1079 m (0.32%) and 1261 m (0.29%) altitudes were placed into the same highest group. In some specific studies, it was indicated that chemical metabolic profiles of plants belonging to Lamiaceae family with pharmacological properties, were affected by abiotic and biotic factors such as ecological conditions, soil profile, weeds, diseases and pests, harvest periods, geographical region and especially altitude^20,21,58^.

Caryophyllene oxide is a compound of oxygenated sesquiterpene group. The greatest caryophyllene oxide ratio (0.35%) was seen at 1079 m altitudes, while the lowest values were obtained seen at 1180 m (0.15%) and 1261 m (0.18%) altitudes. Spathulenol ratios varied between 0.85 and 1.28% and differences were found to be significant (p > 0.001). It was determined that caryophyllene oxide and spathulenol ratios of the samples collected from both low and high altitudes did not exhibit a linear increase or decrease.

Climate parameters vary with the altitudes. Altitude has significant effects on yield levels, essential oil ratios and compositions^59–61^. Temperature, precipitation, relative humidity, day light hours, light intensity and day and night temperature differences change with altitude^31^. Altitude-dependent changes in oxygenated sesquiterpene ratios such as caryophyllene oxide and spathulenol can be explained by prevailing ecological factors of altitudes.

## Conclusion

Present findings clearly revealed that altitude had significant effects on quality criteria of *O. majorana* species (essential oil ratio and secondary metabolite composition), which are largely used in folk medicine and different industries. It was determined that essential oil content of *O. majorana* species with a natural spread in the vegetation, and the linalool component, which is the main active ingredient, decreased with increasing altitudes. Borneol, linalool oxide, trans-linalool oxide, caryophyllene, *a*-humulene, germacrene-D, bicyclogermacrene components yielded higher values at low altitudes. On the other hand, thymol, *a*-terpineol, *a*-terpinene, *cis*-sabinene hydrate, terpinene-4-ol and carvacrol components were positively affected by high altitudes. Abiotic factors, such as climate and soil, affect the amount and composition of essential oil of plants that are cultivated and collected from nature. Within the scope of this study, it was determined that essential oil ratios and components changed with altitudes. Considering the essential oil ratio and the linalool ratio, which is the major component, it was anticipated that *O. majarona* should be collected from low altitudes of the natural flora.

## Data Availability

The datasets generated and analyzed during the current study are not publicly available but are available from the corresponding author on reasonable request.
